# The role of autophagy in periodontal diseases: a bibliometric analysis from 2006 to 2023

**DOI:** 10.3389/fmed.2025.1493459

**Published:** 2025-05-09

**Authors:** Bitong Zhang, Yahui Wang, Haoxiang Chang, Chong Wang, Hong Fan, Xiuyun Ren

**Affiliations:** ^1^Shanxi Medical University School and Hospital of Stomatology, Taiyuan, China; ^2^Shanxi Key Laboratory of Oral Diseases Prevention and New Materials, Taiyuan, China; ^3^Department of Orthodontics, Shanxi Provincial People’s Hospital, The Fifth Clinical Medical College of Shanxi Medical University, Taiyuan, China

**Keywords:** periodontal diseases, autophagy, bibliometric analysis, orthodontic tooth movement, periodontal treatment efficacy

## Abstract

**Background:**

Periodontal disease is a chronic inflammatory condition affecting the supporting structures of the teeth, involving complex interactions between systemic and local immune responses. Autophagy is a tightly regulated cellular process that is responsible for degrading and recycling cellular components, playing a pivotal role in maintaining cellular homeostasis and modulating inflammation in periodontal disease. In recent years, the relationship between these two factors has attracted attention from scholars globally. However, bibliometric analyses in this field are still limited.

**Objectives:**

To analyze the bibliometric trends and research hotspots related to the role of autophagy in periodontal disease.

**Methods:**

Articles and reviews examining the association between periodontal disease and autophagy were retrieved from the Web of Science Core Collection (WOSCC) on 20 June 2024. Bibliometric and knowledge mapping analyses were performed using CiteSpace [6.3. R1 (64-bit) Advanced].

**Results:**

Through a bibliometric analysis of literature published between 2006 and 2023 on the role of autophagy in periodontal disease, 341 relevant studies were identified. The results indicate a steady annual increase in studies on this topic, with a significant upward trend observed post-2015. Keyword analysis identifies “apoptosis,” “Porphyromonas gingivalis,” “oxidative stress,” “inflammation,” “periodontitis,” “osteogenic differentiation,” “cell death,” and “orthodontic tooth movement” as key research hotspots. Collaboration network analysis identifies China as the leading contributor to research in this field. Document co-citation analysis highlights several influential studies examining the “double-edged sword” role of autophagy in periodontal disease, illustrating how autophagy alleviates oxidative stress and inflammation in periodontitis by removing damaged organelles, inhibiting pro-inflammatory mediators, and promoting periodontal tissue repair through the secretion of pro-angiogenic cytokines. However, excessive autophagy may lead to apoptosis when cellular stress surpasses the repair capacity. This study identifies key trends and research hotspots in autophagy and periodontal disease, underscoring the importance of international collaboration and high-impact journals for advancing the field and guiding future research.

**Conclusion:**

Recent studies indicate that autophagy has emerged as a critical mediator with dual roles in periodontal disease. Therefore, early control of periodontal inflammation, along with the exploration of how to harness the protective functions of autophagy, may provide future research directions for managing periodontal disease.

## 1 Introduction

Periodontal Disease (PD) is a chronic inflammatory condition affecting the supporting structures of the teeth, including the gums and alveolar bone. The primary etiological factor is dental plaque, which includes both gingivitis and periodontitis. PD involves complex interactions between systemic and local immune responses, with inflammation playing a central role (1).

Autophagy is a conserved cellular process that maintains homeostasis by degrading and recycling damaged or unnecessary cellular components (2). As a protective mechanism, it alleviates oxidative stress by removing dysfunctional organelles, particularly mitochondria, and modulates inflammatory responses by inhibiting the release of pro-inflammatory mediators and suppressing inflammasome activation (3). Furthermore, autophagy contributes to tissue repair by promoting the secretion of pro-angiogenic cytokines in response to inflammatory conditions. However, under prolonged or excessive stress, dysregulated autophagy may induce apoptosis (4), reflecting a dynamic balance between these two cell death pathways.

In the context of periodontal disease, autophagy exerts multiple protective effects. It contributes to the clearance of pathogenic microorganisms, such as Porphyromonas gingivalis, alleviates oxidative stress by removing dysfunctional mitochondria and reducing reactive oxygen species (ROS), and modulates inflammatory responses by limiting the release of pro-inflammatory cytokines and inhibiting inflammasome activation (5). Additionally, autophagy prevents excessive apoptosis and promotes periodontal tissue regeneration by enhancing the survival and differentiation of periodontal ligament stem cells (PDLSCs) and stimulating the secretion of pro-angiogenic factors, such as angiopoietin (Ang) and basic fibroblast growth factor (bFGF) (6). These mechanisms emphasize autophagy’s pivotal role in preserving periodontal health and highlight its potential as a therapeutic target for mitigating tissue destruction and facilitating repair in periodontitis. However, under prolonged or intense stress, dysregulated or excessive autophagy may shift toward apoptosis, highlighting the need to maintain autophagic balance for periodontal homeostasis (7, 8). Autophagy is not limited to periodontal health; it is widely involved in various biological processes and is closely associated with inflammatory responses. Dysregulation of autophagy has been associated with a range of diseases, including autoimmune disorders, cancer, diabetes, and oral diseases (9).

Bibliometrics is a field that applies mathematical and statistical methods to analyze books, articles, and other scholarly publications. Its purpose is to assess and track the quantity and quality of scientific activities, as well as to understand the structure, development, and trends of academic research (10). In recent decades, bibliometrics has been extensively applied in medical research, including studies on PD, cell death, cancer, COVID-19, and metabolic diseases. However, bibliometric studies focusing on autophagy have predominantly concentrated on cancer, while research on the role of autophagy in periodontal diseases remains relatively limited.

This study aims to conduct a comprehensive bibliometric analysis of the role of autophagy in PD. This study will summarize the knowledge base, research hotspots, and development trends in the field, providing a strong foundation for future research. Elucidating the role of autophagy in PD could significantly influence the diagnosis and treatment of these conditions, thus advancing clinical practice. This will not only assist researchers and experts in gaining a deeper understanding of the relationship between autophagy and periodontal diseases, but also ultimately benefit PD patients by improving treatment outcomes and quality of life.

## 2 Methods

### 2.1 Data source and search strategy

This study utilized the Web of Science Core Collection (WOSCC) database^1^ as the data source, which is widely regarded as the premier database for bibliometric analysis (11). The search query employed was “TS = (“periodontal disease” OR periodont* OR gingiv* OR “tooth loss” OR “tooth migration” OR “tooth mobility”) AND TS = (autophagy).” The search query used was “TS = (“periodontal disease” OR periodont* OR gingiv* OR “tooth loss” OR “tooth migration” OR “tooth mobility”) AND TS = (autophagy).” All retrieved documents were independently assessed by two reviewers (HXC and YHW) based on titles, abstracts, and keywords to exclude studies unrelated to PD and AS. In the event of disagreement between the two reviewers, the decision of a third reviewer (QXJ) was considered final. To minimize bias due to database updates, the literature search was conducted on a single day (2024-06-20).

### 2.2 Inclusion criteria

Publications that met the following criteria were included in the subsequent analysis, while all other articles were excluded:

(1) Articles and review papers related to the fields of PD and autophagy;
(2) Articles and review papers written in English;
(3) Articles and review papers published between 2006 and 2023.

### 2.3 Exclusion criteria

Non-English publications, publication types other than articles and review papers, and publications published before 2006 or after 2023 were excluded from the bibliometric analysis.

### 2.4 Data collection and data analysis

The retrieved papers were exported as “Full Record and Cited References” and saved as “Plain Text.” These files were also named “download_.txt.” Microsoft Office Excel 2019 was used for managing and analyzing annual publications. We also used CiteSpace [6.3. R1 (64-bit) Advanced] to analyze these data and visualize scientific knowledge maps.

CiteSpace, developed by Chaomei Chen, is a Java-based citation visualization tool that provides an experimental platform for exploring new ideas and comparing existing methods. It is one of the most widely used tools for visualization analysis in bibliometrics. CiteSpace can analyze underlying literature from multiple perspectives, identify research hotspots and trends in specific fields, and present these findings visually. Knowledge maps generated by CiteSpace assist researchers in intuitively understanding research hotspots and their evolution, and in predicting research and development trends in areas of interest (12).

## 3 Results

### 3.1 General information

**FIGURE 1:**
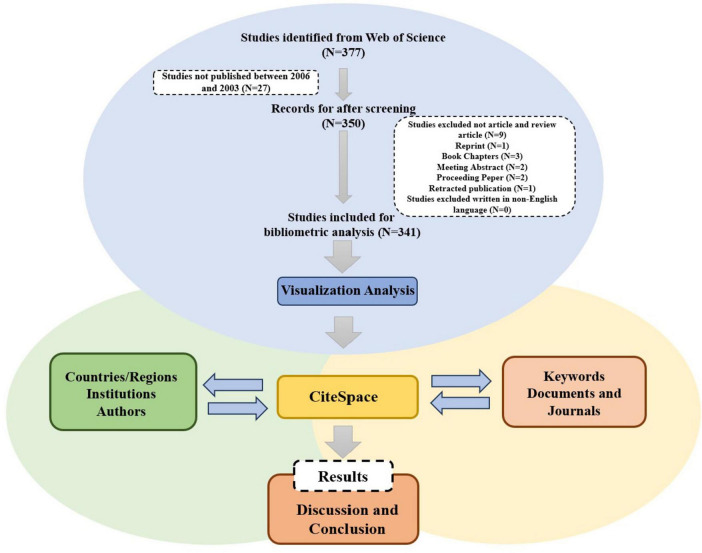
Flowchart of the study methodology.

**FIGURE 2:**
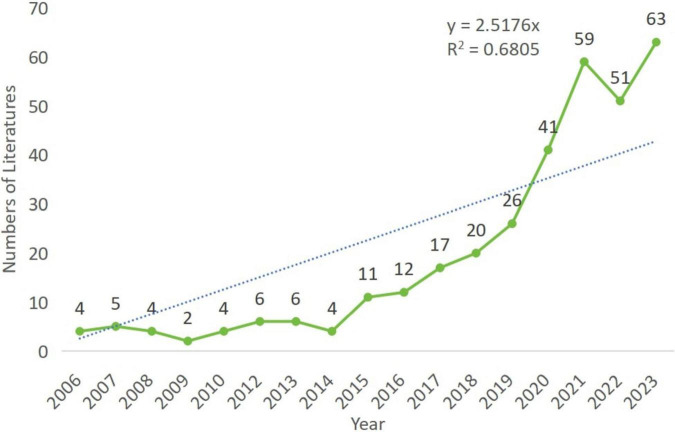
The number of published studies over time.

A total of 377 publications were retrieved from the literature search. After screening, 341 publications were included in the subsequent bibliometric analysis, consisting of 284 research articles (284/341, 83.3%) and 57 review articles (57/341, 16.7%) (Figure 1). From 2000 to 2014, the annual number of publications in this field remained below ten. However, since 2015, there has been a significant increase in the annual number of publications (R^2^ = 0.6805), peaking at 63 papers in 2023 (Figure 2). As shown in Figure 2, the period from 2015 to 2018 saw a rapid increase in publication volume, while from 2019 to 2021, there was exponential growth in the number of publications, which remained high in 2022 and 2023. This trend indicates growing scholarly interest in the relationship between periodontal disease (PD) and autophagy (As) since 2006. From 2015 to 2023, research activity consistently remained high, reflecting the sustained attention of the international academic community in this field.

Overall, research on the role of autophagy in periodontal disease has undergone several significant growth phases over the past 18 years, particularly the rapid increase after 2019, reflecting a marked rise in academic interest in this area. The peak in 2023, with 63 publications, underscores the current high level of research activity.

The increasing annual trend suggests that research on the association between autophagy and periodontal disease is gaining greater recognition, and that additional research findings are likely to emerge, providing stronger scientific evidence for the prevention and treatment of related diseases.

### 3.2 Cooperation network analysis

#### 3.2.1 Distribution of countries/regions

**FIGURE 3:**
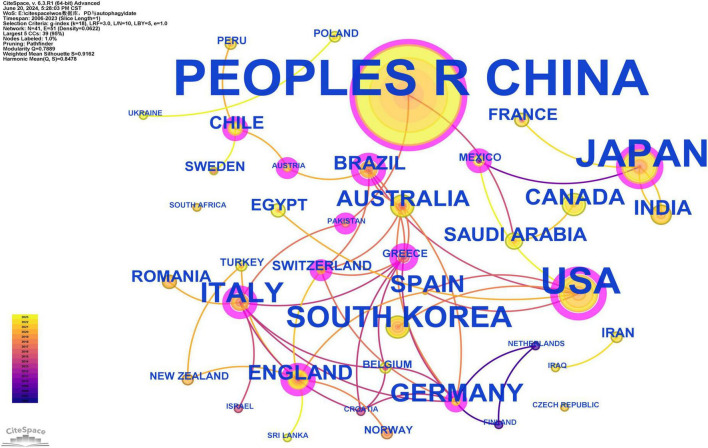
Cooperation network map of countries/regions. After standardizing the sample literature (341 publications), the data were imported into the software CiteSpace for analysis of the selected database, with the time period set from 2006 to 2023. The analysis item was set to “Country,” and a co-occurrence map of countries corresponding to studies on the role of autophagy in PD was generated. The size of the nodes represents the number of publications, and the connections between nodes represent collaborations. The different colors represent the years of collaboration. The outermost purple rings indicate the levels of betweenness centrality, with nodes of high centrality considered as key points in the research field [g-index (k = 18), *N* = 41 (number of network nodes), E = 51 (number of connections), Density = 0.0622 (network density)].

The 341 publications originated from 41 countries, with China contributing over half of the publications (186/341, 54.55%), followed by Japan (*n* = 48) and the United States (*n* = 42) (Supplementary Table 1). This highlights China’s significant influence in this field. Additionally, Brazil achieved the highest Betweenness Centrality (BC) (0.68), reflecting a high level of collaboration with other countries (Supplementary Table 1). The visualization of country/region collaboration reveals that Brazil’s close collaborators are primarily developed countries, including the United States, Germany, Austria, Switzerland, and Australia (Figure 3).

#### 3.2.2 Distribution of institutions

**FIGURE 4:**
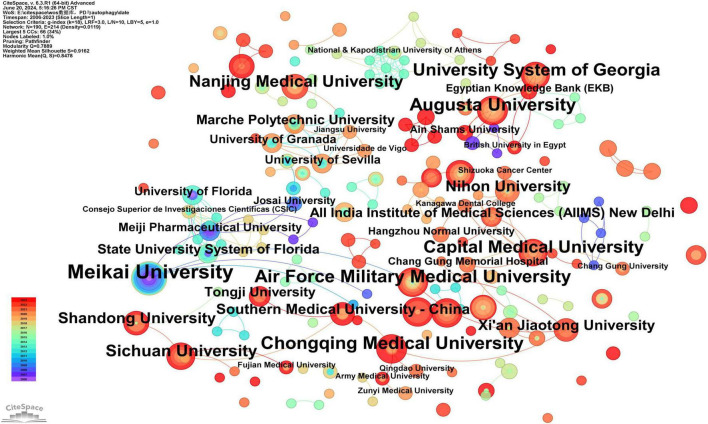
Cooperation network map of institutions. After standardizing the sample literature (341 publications), the data were imported into the software CiteSpace for analysis of the selected database, with the time period set from 2006 to 2023. The analysis item was set to “Institution,” and a co-occurrence map of institutions corresponding to studies on the role of autophagy in PD was generated. The size of the nodes represents the number of publications, and the connections between nodes represent collaborations. The different colors represent the years of collaboration. The outermost purple rings indicate the levels of betweenness centrality, with nodes of high centrality considered as key points in the research field [g-index (k = 18), *N* = 190 (number of network nodes), E = 214 (number of connections), Density = 0.0119 (network density)].

Authors from 190 institutions have contributed to research in this field. Meikai University has the highest number of publications (*n* = 14) (Supplementary Table 2). Notably, none of the institutions have a betweenness centrality (BC) exceeding 0.1, reflecting limited collaboration among institutions in this field (Supplementary Table 2). As shown in Figure 4, some institutions have emerged as key contributors to research on the role of autophagy in PD. The largest node represents Meikai University, followed by Chongqing Medical University, Zhejiang University, Air Force Military Medical University, Augusta University, Peking University, Capital Medical University, University System of Georgia, Sun Yat-sen University, and Sichuan University.

Further analysis of Figure 4 reveals two key characteristics of research institutions in this field:

(1) Among the top 10 institutions by publication volume, seven are from China, highlighting the significant influence of Chinese institutions in this field, consistent with the results presented in Figure 3.
(2) The geographical distribution of research institutions in this field is broad, with significant research interest across various regions worldwide. For example, research is actively conducted in Beijing, Sichuan, Xi’an, and Guangdong in China, as well as in the United States, Japan, Germany, the United Kingdom, and Australia, demonstrating the global importance of this research topic.

#### 3.2.3 Authors and co-cited authors

**FIGURE 5:**
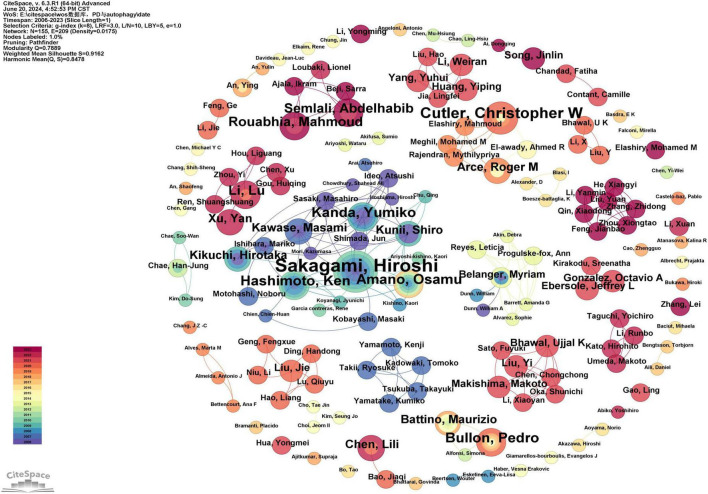
Cooperation network map of authors. After standardizing the sample literature (341 publications), the data were imported into the software CiteSpace for analysis of the selected database, with the time period set from 2006 to 2023. The analysis item was set to “Author,” and a co-occurrence map of authors corresponding to studies on the role of autophagy in PD was generated. The size of the nodes represents the number of publications by each author, and the connections between nodes represent collaborations. The different colors represent the years of collaboration [g-index (k = 8), *N* = 155 (number of network nodes), E = 209 (number of connections), Density = 0.0175 (network density)].

A total of 155 authors contributed to publications on the role of autophagy in periodontal disease (PD). The most prolific author is Hiroshi Sakagami, with 12 publications, followed by Christopher W. Cutler (*n* = 7), Ken Hashimoto (*n* = 6), Pedro Bullon (*n* = 6), and Osamu Amano (*n* = 6) (Supplementary Table 3). As indicated in Supplementary Table 3, none of the authors have a betweenness centrality (BC) exceeding 0.1, reflecting limited collaboration among authors, with research often conducted in small groups or independently. This phenomenon is not uncommon in scientific research, particularly in specialized fields, where authors tend to collaborate more with familiar colleagues or specific research teams. As shown in Figure 5, early research in this field was conducted by a few authors, including Hiroshi Sakagami, Ken Hashimoto, Osamu Amano, Yumiko Kanda, Masami Kawase, and Hirotaka Kikuchi. Mid-period research was led by authors such as Christopher Cutler and Roger M. Arce. Recent research has been carried out by authors such as Abdelhabib Semlali and Mahmoud Rouabhia. It is evident that collaboration among authors such as Hiroshi Sakagami, Ken Hashimoto, and Osamu Amano has been relatively close, forming a significant scale and exerting great influence in this field. Additionally, new authors have published their first papers in this field each year, demonstrating the ongoing appeal and vitality of this research area (Figure 5).

**FIGURE 6:**
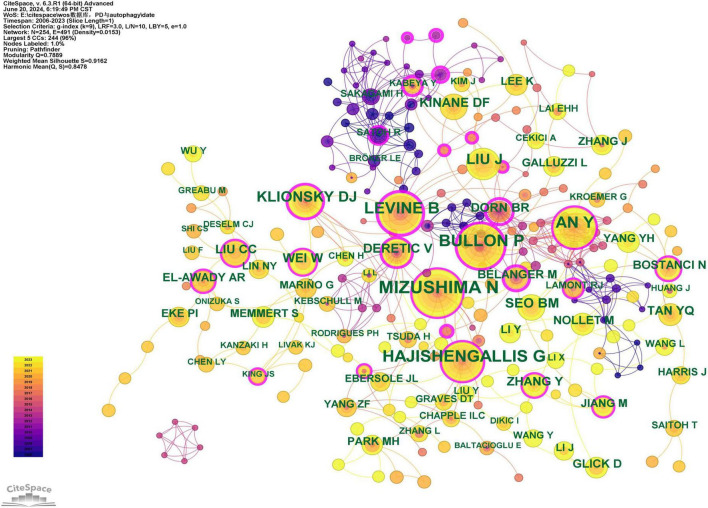
Co-citation network map of authors. After standardizing the sample literature (341 publications), the data were imported into the software CiteSpace for analysis of the selected database, with the time period set from 2006 to 2023. The analysis item was set to “Author Co-citation Network,” and a co-citation map of authors corresponding to studies on the role of autophagy in PD was generated. The size of the nodes represents the number of citations each author received, and the connections between nodes represent instances of being co-cited in a single article. The outermost purple rings indicate the levels of betweenness centrality, with nodes of high centrality considered as key points in the research field. The different colors represent the years of citation [g-index (k = 9), *N* = 254 (number of network nodes), E = 491 (number of connections), Density = 0.0153 (network density)].

Co-citation refers to two or more authors being cited simultaneously by one or more papers. As shown in Supplementary Table 4, the five most co-cited authors have been cited more than 50 times each. The most frequently co-cited author is N. Mizushima (*n* = 78), followed by P. Bullon (*n* = 64), G. Hajishengallis (*n* = 57), B. Levine (*n* = 54), and Y. An (*n* = 53). Furthermore, 31 authors have a BC exceeding 0.10 (see Supplementary Table 4), with B.R. Dorn (0.77) having the highest BC, followed by M. Bélanger (0.52), Y. Kabeya (0.46), T. Adachi (0.42), B. Levine (0.41), and Y.C. Wu (0.41). High BC values indicate their significant bridging roles. Figure 6 presents the author collaboration network. Authors with high centrality are highlighted with purple rings, signifying their crucial bridging roles within the research network. In contrast, some authors exhibited a tendency to collaborate primarily with specific individuals. Correspondingly, their betweenness centrality values were equal to or less than 0.01, suggesting limited influence in connecting different research clusters and indicating a potential need for broader collaboration in future research endeavors.

### 3.3 Keyword analysis

**FIGURE 7:**
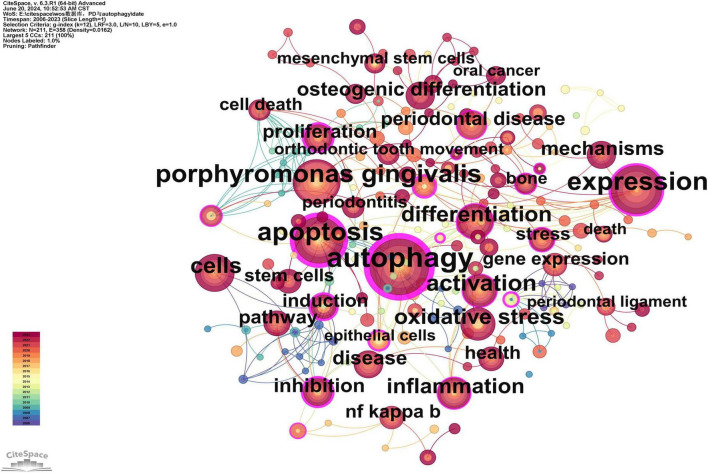
Keywords co-occurrence network map. After standardizing the sample literature (341 publications), the data were imported into the software CiteSpace for analysis of the selected database, with the time period set from 2006 to 2023. The analysis item was set to “Keyword,” and a co-occurrence map of keywords corresponding to studies on the role of autophagy in PD was generated. The size of the nodes represents the frequency of the keywords, and the connections between nodes represent the co-occurrence of different keywords. The different colors represent the years of collaboration. The outermost purple rings indicate the levels of betweenness centrality, with nodes of high centrality considered as key points in the research field [g-index (k = 12), *N* = 211 (number of network nodes), E = 358 (number of connections), Density = 0.0162 (network density)].

Keywords serve as concise summaries of articles, and their analysis facilitates the identification of research hotspots and emerging trends. Therefore, a visualization map of keywords was presented (Figure 7). A total of 211 keywords were identified, with the five most frequent keywords being: autophagy (*n* = 94), expression (*n* = 66), apoptosis (*n* = 64), Porphyromonas gingivalis (*n* = 57), and activation (*n* = 39). The high frequency of these keywords suggests that they are the primary focus of research (Supplementary Table 5). Additionally, the five keywords with the highest betweenness centrality (BC) are: autophagy (0.85), apoptosis (0.63), expression (0.28), epithelial cells (0.26), and adenosine triphosphate (0.25). The high centrality of these keywords suggests that they play key bridging roles in the research network, connecting a wide range of other research topics (Supplementary Table 5).

**FIGURE 8:**
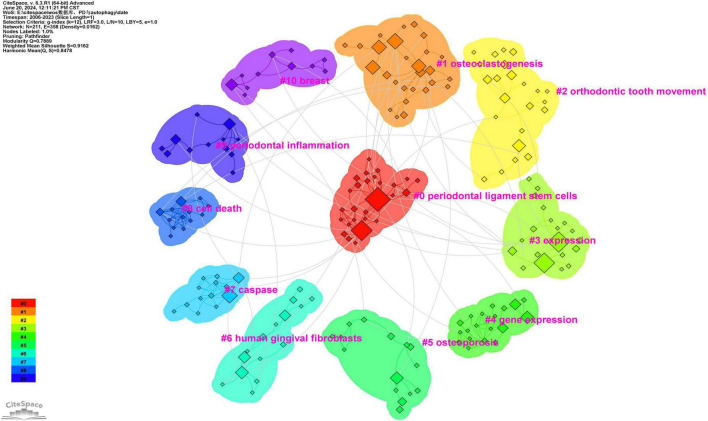
Keywords cluster network map.

To better reflect the research hotspots and trends in this field, we used CiteSpace software to generate a cluster network map (Figure 8). As shown in Figure 8, a total of ten clusters were identified: “periodontal ligament stem cells (#0),” “osteoclastogenesis (#1),” “orthodontic tooth movement (#2),” “expression (#3),” “gene expression (#4),” “osteoporosis (#5),” “human gingival fibroblasts (#6),” “caspase (#7),” “cell death (#8),” “periodontal inflammation (#9)” and “breast (#10)” (Figure 8).

Cluster #0 encompasses keywords such as periodontal ligament stem cells, macrophages, types of cell death, mitophagy, and cell cycle. Analysis of these keywords reveals that this cluster primarily centers on the study of cellular mechanisms in periodontal disease, specifically regarding periodontal ligament stem cells, their role in immune regulation, and their involvement in cell death processes, such as mitophagy and cell cycle regulation.

Cluster #1 encompasses keywords such as osteoclastogenesis, regeneration, mTOR, osteogenesis, and macrophages. Analysis of these keywords reveals that this cluster primarily centers on bone remodeling processes in periodontal disease, particularly osteoclastogenesis and osteogenesis, with a strong emphasis on the role of autophagy and the mTOR pathway in regulating these processes, as well as macrophage involvement in bone homeostasis.

Cluster #2 encompasses keywords such as orthodontic tooth movement, periodontal ligament, periodontitis, mechanical stress, and beclin-1. Analysis of these keywords reveals that this cluster primarily centers on the effects of mechanical stress on periodontal tissues, specifically in the context of orthodontic tooth movement and periodontitis, and the role of autophagy in the response to these stresses, with particular emphasis on beclin-1 in the initiation of autophagy.

Cluster #3 encompasses keywords such as expression, Porphyromonas gingivalis, degradation, tissue, and survival. Analysis of these keywords reveals that this cluster primarily centers on the molecular mechanisms of gene expression in response to Porphyromonas gingivalis infection in periodontal tissues, with particular emphasis on how autophagy mediates pathogen clearance, tissue degradation, and cell survival in periodontal disease.

Cluster #4 encompasses keywords such as osteoporosis, gold nanoparticles, osteogenic differentiation, Mycobacterium tuberculosis, and pathogenesis. Analysis of these keywords reveals that this cluster primarily explores the relationship between osteogenic differentiation, bone diseases such as osteoporosis, and the potential therapeutic application of gold nanoparticles in periodontal tissue regeneration through autophagic pathways.

Cluster #5 encompasses keywords such as human gingival fibroblasts, non-coding RNA, human gingival fibroblasts, and experimental periodontitis. Analysis of these keywords reveals that this cluster primarily examines the role of gingival fibroblasts and non-coding RNA in regulating autophagy during periodontitis, focusing on fibroblast function, tissue regeneration, and the role of non-coding RNA in modulating inflammation and autophagy in periodontal disease.

Cluster #6 encompasses keywords such as human gingival fibroblasts, non-coding RNA, experimental periodontitis, and fibroblast. Analysis of these keywords reveals that this cluster primarily examines the role of human gingival fibroblasts in periodontal disease, particularly concerning the influence of non-coding RNAs on inflammation and tissue repair mechanisms.

Cluster #7 encompasses keywords such as caspase, DNA fragmentation, apoptosis, and anticancer agents. Analysis of these keywords reveals that this cluster primarily investigates apoptosis and caspase activation in periodontal disease, with a focus on cell death pathways and potential therapeutic interventions, including anticancer agents that regulate cell survival.

Cluster #8 encompasses keywords such as cell death, oral squamous cell carcinoma, virus, CCAAT/enhancer-binding protein homologous protein, and glucose-regulated protein 78. Analysis of these keywords reveals that this cluster primarily investigates the interaction between cell death mechanisms and oral squamous cell carcinoma (OSCC), focusing on viral infections and stress proteins involved in both cancer progression and periodontal tissue damage.

Cluster #9 encompasses keywords such as periodontal inflammation, alveolar bone, ATG5, and mechanical stress. Analysis of these keywords reveals that this cluster primarily investigates the role of autophagy, specifically ATG5, in regulating periodontal inflammation and alveolar bone remodeling, influenced by mechanical stress during orthodontic tooth movement.

Cluster #10 encompasses keywords such as breast, cigarette smoke, esophageal SCC, and activation. Analysis of these keywords reveals that this cluster primarily examines the systemic effects of cigarette smoke, particularly its role in cancer progression and periodontal inflammation, suggesting a cross-talk between oral health and cancer through shared molecular pathways.

**FIGURE 9:**
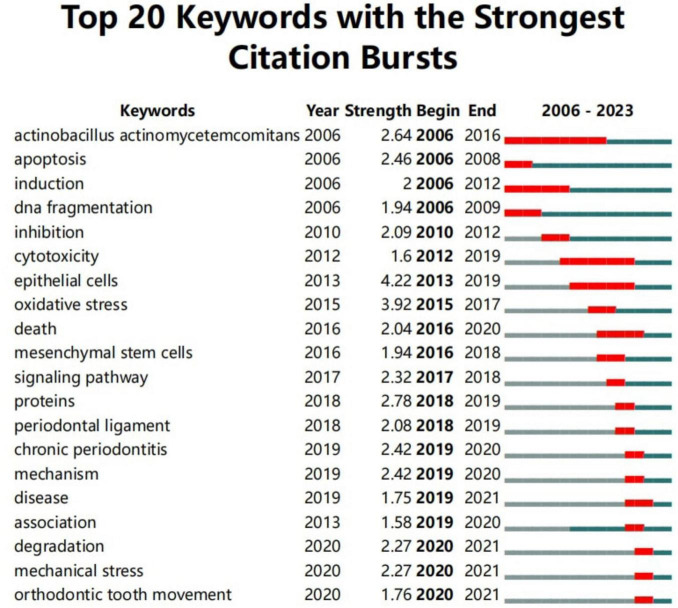
Top 20 keywords with the strongest citation bursts. The red line represents years when keywords burst, and the blue line indicates years when keywords were used less frequently. Burst strength reflects the occurrence times of keywords in a certain period. Each keyword burst lasts for a minimum duration of 1 year.

Burst keyword analysis examines the temporal distribution of keywords to identify frontier areas and development trends in a discipline, detecting keywords with high change rates and rapid growth. As shown in Figure 9, the keyword Actinobacillus actinomycetemcomitans had the longest burst duration, while “epithelial cells” had the highest burst intensity. Furthermore, “degradation,” “mechanical stress,” and “orthodontic tooth movement” have emerged as the latest burst keywords since 2020.

A time zone map consists of a series of vertical bands representing time zones, illustrating the evolving knowledge map from a temporal perspective. It clearly demonstrates the relationships among documents and their updates. The time zones are arranged chronologically from left to right, aligning the research front with its knowledge base. The “Time Zone” feature in CiteSpace not only displays citation keyword information but also reveals cited document information in the background data, emphasizing the connection between the research front and its knowledge base.

**FIGURE 10:**
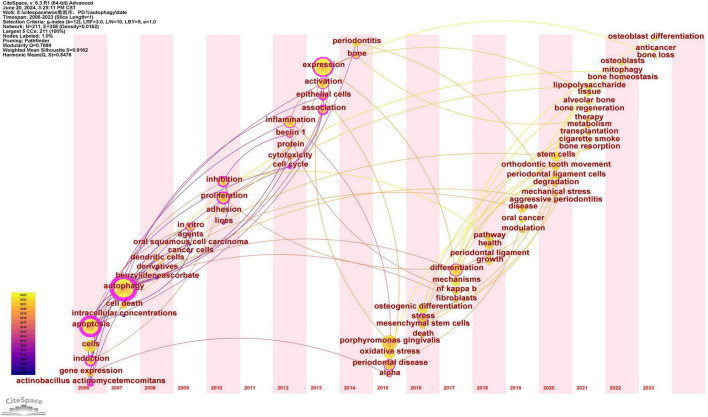
Keyword time zone map. In the figure, the nodes represent keywords, with node size indicating the frequency of the keywords and color representing the time of keyword appearance. The lines represent the co-occurrence relationships between keywords.

After standardizing the sample literature (341 publications), the data were imported into CiteSpace for analysis, with the period set from 2006 to 2023 and a time slice of 1 year. The analysis item was set to “Keyword,” generating a keyword co-occurrence map. Using the Time Zone feature, a time zone view of the role of autophagy in PD from 2006 to 2023 was created, as shown in Figure 10. Figure 10 clearly illustrates the evolution of research hotspots and frontiers in this field, offering important insights into the dynamics of the role of autophagy in PD.

According to Figure 10, research in this field can be broadly divided into three stages:

Early research (2006–2010), with key keywords: apoptosis, gene expression, autophagy, cell death, Actinobacillus actinomycetemcomitans, oral squamous cell carcinoma, inhibition. This period focused on fundamental biological mechanisms, including the role of autophagy in apoptosis, gene expression, and DNA damage repair. Some studies also explored the role of autophagy in pathogen infection and cancer therapies.

Mid-term research (2011–2017), with key keywords: cell cycle, Beclin 1, inflammation, osteogenic differentiation, periodontitis, oxidative stress, mesenchymal stem cells, pathway, NF-κB. The research focus shifted toward the specific mechanisms of autophagy in periodontal disease and other conditions.

Recent research (2018–2023), with key keywords: osteoblast differentiation, bone loss, bone homeostasis, bone regeneration, therapy, periodontal ligament stem cells, mechanical stress, and cigarette smoke. Recent studies have increasingly focused on the specific applications and regulatory mechanisms of autophagy, including periodontal tissue homeostasis, inflammation control, periodontal tissue destruction, and the impact of environmental factors on autophagy.

### 3.4 Co-cited document and journal

**FIGURE 11:**
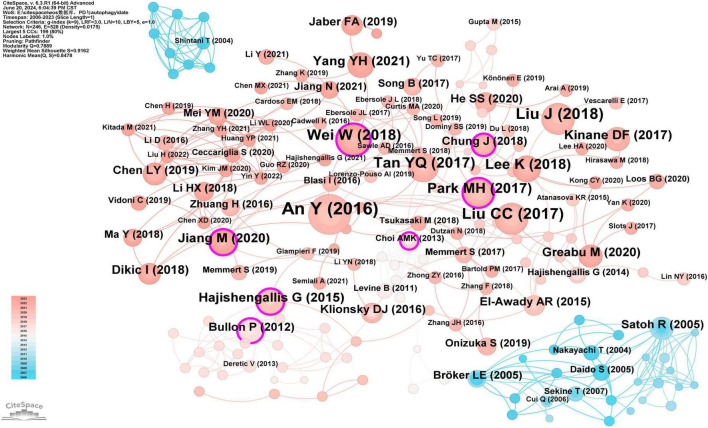
Cooperation network map of documents. After standardizing the sample literature (341 publications), the data were imported into the software CiteSpace for analysis of the selected database, with the time period set from 2006 to 2023. The analysis item was set to “document,” and a co-citation map of documents corresponding to studies on the role of autophagy in PD was generated. The size of the nodes represents the number of citations each document received, and the connections between nodes represent instances of being co-cited in a single article. The different colors represent the years of collaboration. The outermost purple rings indicate the levels of betweenness centrality, with nodes of high centrality considered as key points in the research field [g-index (k = 9), *N* = 246 (number of network nodes), E = 528 (number of connections), Density = 0.0175 (network density)].

Co-citation analysis identifies core literature and research hotspots in a specific field by recognizing frequently co-cited documents. Using CiteSpace software with a g-index of 9, we screened 246 cited documents (Figure 11), with the top five most-cited documents being: An Y, 2016, J Clin Periodontol (*n* = 30) (4), Liu CC, 2017, Front Physiol (*n* = 20) (7), Liu J, 2018, Life Sci (*n* = 19) (13), Wei W, 2018, J Periodontol (*n* = 18) (14), and Tan YQ, 2017, Autophagy (*n* = 17) (9). Additionally, seven articles had a BC greater than or equal to 0.10, namely Park MH, 2017, Mol Oral Microbiol (0.15) (5), Bullon P, 2012, BMC Med (0.14) (15), Wei W, 2018, J Periodontol (0.13) (14), Hajishengallis G, 2015, Nat Rev Immunol (0.11) (16), Chung J, 2018, J Periodontol (0.11) (17), Choi AMK, 2013, N Engl J Med (0.11) (18), and Jiang M, 2020, Oral Dis (0.10) (19) (Supplementary Table 6). The high citation counts and high BC values of these documents indicate their widespread recognition within the field.

**FIGURE 12:**
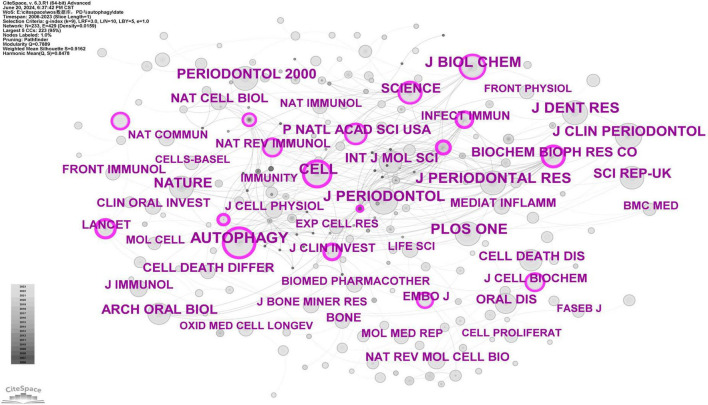
Cooperation network map of journals. After standardizing the sample literature (341 publications), the data were imported into the software CiteSpace for analysis of the selected database, with the time period set from 2006 to 2023. The analysis item was set to “journal,” and a co-citation map of journals corresponding to studies on the role of autophagy in PD was generated. The size of the nodes represents the number of citations each journal received, and the connections between nodes represent instances of being co-cited in a single article. The different colors represent the years of collaboration. The outermost purple rings indicate the levels of betweenness centrality, with nodes of high centrality considered as key points in the research field [g-index (k = 9), *N* = 233 (number of network nodes), E = 429 (number of connections), Density = 0.0159 (network density)].

Journal co-citation analysis identifies core journals and communication channels within a specific research field by analyzing the frequency with which journals are co-cited. Using CiteSpace software with a g-index of 9, we screened a total of 233 journals (Figure 12). According to Figure 12, core journals in periodontology, such as J Periodontol, J Dent Res, J Periodontal Res, J Clin Periodontol, and Periodontol 2000, are frequently cited, highlighting the significance of autophagy research in periodontal diseases. The most cited journal is Autophagy (*n* = 208) (Supplementary Table 7), a core journal in the field, confirming the critical role of autophagy in periodontal disease research. High-impact journals such as Cell, Nature, Science, and Lancet are frequently cited, demonstrating the substantial scientific value and wide influence of related research.

## 4 Discussion

### 4.1 General information

This study systematically analyzed the role of autophagy in periodontal disease (PD) using bibliometric methods. This analysis not only revealed research hotspots and trends but also offered valuable insights into future research directions. From 2006 to 2023, research on the role of autophagy in PD exhibited significant growth, particularly after 2015 (Figure 2). The increase in research volume reflects the academic community’s growing attention to the role of autophagy in PD, likely due to the increasing recognition of autophagy’s critical function in regulating chronic inflammation.

Scientific collaboration is defined as the joint effort of researchers to generate new scientific knowledge. Scientific collaboration occurs at various levels, including individual, institutional, and international collaboration. When assessing the importance of nodes in a collaboration network, both social connectivity and academic output are critical, with betweenness centrality (BC) being a key indicator of social connectivity. If a node’s BC exceeds 0.10, it indicates that the node is central, relatively important, and highly influential in research (20). At the national level, as shown in Figure 3 and Supplementary Table 1, China (*n* = 186), Japan (*n* = 48), and the United States (*n* = 42) play key roles in this field. Notably, China leads in the number of publications, accounting for more than half of the total (54.55%), with a BC greater than 0.10, demonstrating its significant influence in this research area. Brazil achieved the highest BC (0.57) in the national cooperation analysis (Supplementary Table 1), far surpassing China (0.15), which may be attributed to Brazil’s economic development and recent improvements in public health (21). The top three institutions in terms of publication volume are Meikai University (*n* = 14), Chongqing Medical University (*n* = 10), and Zhejiang University (*n* = 10), with Xi’an Jiaotong University having the highest BC (0.03) (Supplementary Table 2). Meikai University is located in Japan, and the other three institutions are based in China, further demonstrating the significant influence and leadership of China and Japan in this field. In our analysis, Sakagami, Hiroshi published the most articles (*n* = 14), followed by Cutler, Christopher W (*n* = 7), Hashimoto, Ken (*n* = 6), Bullon, Pedro (*n* = 6), and Amano, Osamu (*n* = 6) (Supplementary Table 3). There is limited extensive collaboration among authors, who tend to collaborate with familiar colleagues or specific research teams, such as the close collaboration between Sakagami, Hiroshi, Hashimoto, Ken, and Amano, Osamu, among others. To address current limitations in collaboration among authors and institutions and to foster more extensive and deeper partnerships within the field, we propose the following recommendations. First, the regular organization of multidisciplinary workshops and thematic conferences can facilitate cross-disciplinary communication and promote the integration of diverse academic perspectives. Second, international collaboration should be strengthened by encouraging research teams to apply for international funding programs, establish joint laboratories abroad, and actively engage in international academic exchanges. Third, establishing inter-institutional cooperation platforms at regional and national levels is essential for the efficient sharing of data, methodologies, and research resources. Finally, closer collaboration between academia and industry should be fostered by aligning industrial demands with academic expertise, thus accelerating the translation of scientific findings into practical applications. Overall, enhancing international and cross-sector collaboration has the potential to bridge disciplinary gaps, overcome institutional barriers, and create a positive feedback loop that drives the advancement of high-quality scientific research.

The top 10 co-cited authors were each cited more than 20 times, and seven authors played significant bridging roles (BC > 0.1), indicating their influence as key promoters of knowledge dissemination and interdisciplinary collaboration (Supplementary Table 4). They play pivotal roles in the academic network, making substantial contributions to the development and innovation of this research field.

Journal co-citation analysis reveals that the top 10 cited journals in this field are Autophagy (IF = 14.6), J Periodontol (IF = 4.2), J Dent Res (IF = 5.7), J Biol Chem (IF = 4), J Periodontal Res (IF = 3.4), Cell (IF = 45.5), PLOS ONE (IF = 2.9), Nature (IF = 50.5), J Clin Periodontol (IF = 5.8), and Periodontol 2000 (IF = 17.5) (Supplementary Table 7). J Periodontol, J Dent Res, J Periodontal Res, J Clin Periodontol, and Periodontol 2000 are leading journals in periodontology; Autophagy is a prominent journal in the field of autophagy; Nature and Cell are top-tier journals in the general scientific domain. The high citation frequency of these journals suggests that the role of autophagy in PD is an interdisciplinary topic with substantial scientific value and broad influence.

### 4.2 Knowledge base

Co-citation analysis measures the degree of association between academic papers. A knowledge base refers to a collection of commonly cited references (8). This study includes the five most co-cited papers in the field of “the role of autophagy in PD” (Supplementary Table 6). The most cited article is “Increased autophagy is required to protect periodontal ligament stem cells from apoptosis in the inflammatory microenvironment” by An Y et al., published in 2016 in J Clin Periodontol (4). This study utilized immunohistochemistry to detect LC3 expression in periodontal ligament (PDL) tissues from patients with periodontitis (*n* = 20) and healthy controls (*n* = 20). Additionally, to further investigate the mechanism of autophagy, the study conducted cell experiments with PDLSCs divided into three groups: healthy PDLSCs (H-PDLSCs), periodontitis PDLSCs (P-PDLSCs), and inflammation-simulated PDLSCs (I-PDLSCs). Autophagy levels in PDLSCs were measured by qRT-PCR and Western blotting. LC3-positive spots were identified by immunofluorescence, and autophagic vesicles (AVs) were observed using transmission electron microscopy. Results: Elevated levels of autophagy gene expression and autophagosome formation were observed in the PDL tissues of periodontitis patients. Increased protein levels of LC3, Beclin-1, Atg7, and Atg12 were found in P-PDLSCs and I-PDLSCs. LC3-positive spots and AVs were identified in P-PDLSCs and I-PDLSCs. Activation of autophagy may protect PDLSCs from apoptosis. Conclusion: The study suggests that regulating autophagy in P-PDLSCs may offer a novel therapeutic strategy for enhancing periodontal treatment.

The second article, “The Role of Reactive Oxygen Species and Autophagy in Periodontitis and Their Potential Linkage,” by Liu CC et al., was published in 2017 in Front Physiol (7). This review examines the role of the imbalance between reactive oxygen species (ROS) and antioxidant defenses in the pathogenesis of periodontitis. The main points highlight that ROS and autophagy play crucial roles in periodontitis. Elevated ROS levels induce oxidative stress and periodontal tissue damage, while autophagy regulates cell fate, promoting both cell death and preventing apoptosis. The specific roles of autophagy in periodontitis include bacterial clearance, regulation of immune responses, and inhibition of periodontal cell apoptosis.

The third article, “Lipopolysaccharide from Porphyromonas gingivalis promotes autophagy of human gingival fibroblasts through the PI3K/Akt/mTOR signaling pathway,” by Liu J et al., was published in 2018 in Life Sci (13). This study treated human gingival fibroblasts (HGFs) with lipopolysaccharide (LPS) from Porphyromonas gingivalis (P.g) and observed autophagosome formation and the conversion of microtubule-associated protein light chain 3 (LC3)-II using both morphological and quantitative methods to evaluate whether the PI3K/Akt/mTOR signaling pathway mediates LPS-induced autophagy in HGFs. Main results: Increased autophagy was observed in LPS-treated cells compared to controls, with significantly higher expression of LC3-II and ATG5 mRNA and proteins, as well as more LC3 puncta and autophagosomes observed via immunofluorescence microscopy and transmission electron microscopy. LPS-treated cells exhibited significantly decreased expression of p-PI3K, p-Akt, and mTOR. Experiments with the PI3K activator 740Y-P and the Akt-specific activator SC79 confirmed that inhibition of the PI3K/Akt/mTOR pathway is associated with LPS-induced autophagy. The autophagy inhibitor 3-MA increased LPS-induced secretion of TNF-α and IL-1β. Conclusion: LPS from Porphyromonas gingivalis induces autophagy in HGFs by inhibiting the PI3K/Akt/mTOR signaling pathway, and autophagy helps suppress the secretion of inflammatory cytokines induced by LPS.

The fourth article, “Activation of Autophagy in Periodontal Ligament Mesenchymal Stem Cells Promotes Angiogenesis in Periodontitis,” by Wei W et al., was published in 2018 in J Periodontol (14). This study aimed to investigate the ability of PDLSCs to promote angiogenesis within an inflammatory environment and explore the mechanisms underlying abnormal angiogenesis in periodontitis. Healthy PDLSCs (HPDLSCs) and periodontitis PDLSCs (PPDLSCs) were isolated from human periodontal ligament tissues under healthy and inflammatory conditions, respectively, and the expression of autophagy-related proteins and pro-angiogenic cytokines was evaluated by qRT-PCR and Western blot. The angiogenic capacity of endothelial cells was measured using the Matrigel tube formation assay. Autophagy levels in PDLSCs were modulated by rapamycin-induced autophagy and siRNA-mediated inhibition of Beclin-1 expression. Main results: The inflammatory environment increased the expression of autophagy-related and pro-angiogenic factors. Rapamycin-induced autophagy enhanced the angiogenic capacity. Inhibition of autophagy diminished angiogenic capacity. Conclusion: Autophagy promotes angiogenesis in periodontitis by modulating the paracrine function of PDLSCs. Inflammation-related autophagy functions as a switch to modulate the pro-angiogenic potential of PDLSCs, unveiling a new mechanism of abnormal angiogenesis in periodontitis and offering new insights for potential therapeutic strategies.

The fifth article, “Autophagy and its implication in human oral diseases,” by Tan YQ et al., was published in 2017 in Autophagy (9). This study provides a summary of the mechanisms and types of autophagy and their roles in human oral diseases. Key findings: Autophagy is categorized into macroautophagy, microautophagy, and chaperone-mediated autophagy (CMA), each differing in the method of delivery of cytoplasmic components to lysosomes and their physiological functions. Macroautophagy (commonly referred to as autophagy) is the most extensively studied type, involving the formation of double-membrane autophagosomes that encapsulate cellular components for lysosomal degradation. Autophagy plays a “double-edged sword” role in oral cancer, inhibiting tumorigenesis while also promoting tumor growth, with potential applications in anti-cancer therapy for oral squamous cell carcinoma (OSCC). Autophagy plays a critical role in tooth development and the pathogenesis of periapical lesions, initially protecting cell survival and later promoting cell death. In periodontal disease, autophagy regulates inflammation and immune responses by modulating cell death and inhibiting apoptosis in infected cells. Autophagy is essential for Candida albicans infection survival and pathogenicity, aiding bacterial adaptation and survival through vacuole-mediated autophagy. Conclusion: Autophagy plays a significant role in the pathogenesis of oral diseases, exerting protective effects while also having the potential for pathological progression under specific conditions. Future research should focus on exploring the interactions between autophagy, apoptosis, immune responses, and other cellular processes to identify new therapeutic targets and strategies.

These five articles clearly demonstrate that autophagy not only functions as an “inflammation regulator,” controlling the extent of inflammation, but also protects cells from apoptosis. However, excessive inflammation can induce autophagy, thereby promoting cell death. Therefore, early intervention in periodontal inflammation and exploration of how to harness the protective functions of autophagy to manage periodontal disease may constitute promising future research directions.

### 4.3 Research hotspots between autophagy and PD

Keywords provide insight into research hotspots and trends within a specific field. As shown in Supplementary Table 5, the top 30 keywords appeared more than 10 times, representing key research hotspots in the role of autophagy in PD. Representative keywords include apoptosis, Porphyromonas gingivalis, oxidative stress, inflammation, periodontitis, osteogenic differentiation, cell death, and orthodontic tooth movement. Additionally, burst keyword analysis examines the temporal distribution of keywords to identify emerging trends and frontier areas within a discipline, with particular emphasis on those experiencing rapid growth. “Epithelial cells” exhibited the highest burst intensity, while “mechanical stress” and “orthodontic tooth movement” have emerged as the most recent burst keywords since 2020. Based on the previous discussion, the current research hotspots in this field can be summarized as follows.

#### 4.3.1 Alveolar bone remodeling

Autophagy plays a crucial role in alveolar bone remodeling. PDLSCs exhibit significant potential in periodontal tissue repair, and their osteogenic differentiation is essential for the formation of new alveolar bone (22, 23). Recent studies have shown that in human periodontal ligament fibroblasts, upregulation of alkaline phosphatase activity, formation of mineralized nodules, and increased expression of osteogenic-related genes correlate with enhanced autophagic activity (24). Blockade of autophagy inhibits osteogenic differentiation. This suggests that autophagy may impact periodontal tissue regeneration by influencing the osteogenic differentiation of PDLSCs (25). Some studies suggest that gold nanoparticles can enhance osteogenic differentiation of PDLSCs by stimulating autophagic activity (26).

#### 4.3.2 Epithelial cells

Epithelial cells have garnered increasing attention in the field of autophagy and periodontal disease due to their pivotal role in maintaining mucosal barrier integrity and modulating immune responses (27, 28). As primary defenders against pathogens like Porphyromonas gingivalis, epithelial cells utilize autophagy to preserve tight junctions and regulate inflammation. However, Porphyromonas gingivalis can disrupt this defense by inhibiting the ATG5–LC3 pathway, increasing barrier permeability and promoting systemic inflammation via the oral–gut (28). Autophagy acts as a double-edged sword: while it clears pathogens and maintains homeostasis, it may also be hijacked by pathogens for survival (29). These findings raise key questions: How do pathogens specifically manipulate autophagy? What are the temporal dynamics between autophagy and other death pathways like apoptosis and pyroptosis? How do epithelial cells across tissues differ in their autophagic responses? Can therapeutic modulation of autophagy restore barrier function and reduce inflammation? Addressing these questions will deepen our understanding of the pathogen–autophagy–immune network and support the development of precision therapies targeting epithelial cells in periodontal and systemic diseases.

#### 4.3.3 Orthodontic tooth movement

In recent years, the growing number of adult orthodontic patients and increased awareness of periodontal health have significantly heightened research interest at the intersection of autophagy, orthodontics, and periodontitis. Orthodontic force-induced oxidative stress has been shown to activate autophagy, which plays a dual role in regulating inflammation, bone remodeling, and tissue homeostasis (30). Mechanical force applied during orthodontic tooth movement induces a biological response in periodontal tissues, activating autophagy, particularly on the pressure side. Studies have shown that the expression of autophagy-related proteins, such as Beclin-1 and LC3-II, significantly increases in the early stages of OTM, indicating the initiation of the autophagic process (31). Autophagy plays a pivotal role in regulating the inflammatory response. During OTM, periodontal ligament cells subjected to mechanical forces undergo local inflammation and enhanced autophagy. Research indicates that autophagy regulates the inflammatory response, assisting cells in coping with mechanical stress and nutrient deprivation. During OTM, autophagy influences bone remodeling by modulating osteoclast activity. The aggregation of osteoclasts on the pressure side correlates with autophagy activation (32). Studies have shown that excessive autophagy can impair osteoclast recruitment, thereby limiting bone resorption and tooth movement. Additionally, autophagy-activating drugs such as rapamycin can reduce osteoclast numbers, further validating the regulatory role of autophagy in bone remodeling (32, 33). This intricate interplay has given rise to several critical research questions, including how mechanical stress modulates autophagy through signaling pathways such as AMPK/mTOR, the specific role of autophagy in osteoblast function and inflammatory responses, and its potential as a biomarker and therapeutic target in personalized orthodontic treatment. Future research should focus on the spatiotemporal regulation of autophagy, the integration of multiple signaling pathways, and the development of translational strategies to enhance clinical precision and therapeutic efficacy in orthodontic care.

#### 4.3.4 Periodontal treatment

The treatment of periodontal disease is essential for maintaining oral health and preventing tooth loss. Early diagnosis and effective interventions not only help prevent tooth extraction but also substantially improve overall health (34). Traditional periodontal treatments, including scaling and root planning (SRP) and periodontal surgery, aim to remove plaque and calculus from the tooth surface to reduce inflammation and infection, thereby controlling disease progression. However, these methods may cause discomfort in some cases and are often limited in their efficacy for treating advanced periodontal destruction, such as bone loss (35). With advancements in technology, an increasing number of innovative treatments are being incorporated into periodontal disease therapy.

Laser therapy and ultrasonic treatments are two innovations that have gained widespread use in recent years. Laser therapy utilizes precise high-energy laser beams to remove damaged tissue and promote wound healing, providing effective anti-inflammatory benefits with minimal tissue damage (36). Ultrasonic treatment uses high-frequency vibrations to efficiently remove calculus and plaque, minimizing impact on soft tissues and reducing patient discomfort during traditional scaling procedures. These emerging techniques not only enhance treatment efficiency and reduce patient discomfort but also improve overall treatment outcomes (37).

As research progresses, accumulating evidence underscores the innovative potential of autophagy as a therapeutic intervention in periodontal disease management. Autophagy, a natural cellular repair mechanism, plays a pivotal role in clearing damaged organelles and pathogens, thereby maintaining cellular homeostasis (38, 39). In the context of periodontal disease treatment, modulating autophagy pathways can effectively enhance tissue regeneration, mitigate inflammation, and strengthen immune defenses (40, 41). Autophagy regulation holds promise for optimizing periodontal tissue repair and improving the effectiveness of current therapeutic approaches, particularly in tissue regeneration and bone remodeling. Therefore, integrating autophagy modulation into periodontal disease treatment strategies could open the door to novel therapeutic options in this field.

In conclusion, autophagy plays a “double-edged sword” role in periodontal disease (Table 1). Autophagy maintains cellular homeostasis by clearing damaged organelles and proteins while protecting cells from damage by regulating apoptosis and inflammation. In pathological conditions such as periodontitis, the regulatory mechanisms of autophagy play a crucial role in maintaining cellular homeostasis and protecting tissues from damage. However, when the stimulation is excessive or prolonged, autophagy can trigger apoptosis, leading to tissue destruction. These findings offer new perspectives on understanding the role of autophagy in periodontal disease and present potential targets for developing new therapeutic strategies against periodontitis.

**TABLE 1:** Dual roles of autophagy in periodontitis.

Mechanism/process	Protective effects	Harmful effects	References
Pathogen clearance	Autophagy promotes the clearance of periodontal pathogens, such as Porphyromonas gingivalis, thereby mitigating infection and inflammation.	Impaired or insufficient autophagic response can hinder pathogen clearance, thereby elevating the risk of infection.	(8, 29, 42)
Inflammatory regulation	Regulated autophagy modulates cytokine secretion and immune responses, thereby contributing to the maintenance of a balanced inflammatory state and the protection of periodontal tissues.	Dysregulated or excessive autophagy may contribute to chronic inflammation, leading to subsequent tissue damage.	(4, 43)
Cell survival and tissue regeneration	Autophagy promotes the differentiation and survival of periodontal ligament stem cells (PDLSCs), supporting tissue regeneration.	Overactivation of autophagy can impair stem cell function and cause apoptosis, hindering tissue regeneration.	(6)
Promotion of bone tissue repair	Autophagy modulates osteogenic differentiation and mineralization, contributing to alveolar bone regeneration and mitigating bone loss in periodontitis.	Overactivation of autophagy in osteoclasts may promote bone resorption and disrupt bone homeostasis, exacerbating alveolar bone loss.	(29, 40, 41, 44)
Influence on host immune function	Proper autophagy supports immune cell function, aiding in the clearance of pathogens and maintaining periodontal tissue integrity.	Dysregulated autophagy can impair immune surveillance, reducing pathogen clearance and facilitating disease progression.	(29)
Mitochondrial homeostasis	Mitophagy helps maintain mitochondrial function in periodontal cells, promoting cell survival and preventing oxidative stress.	Defective mitophagy can lead to mitochondrial dysfunction, increasing oxidative stress and contributing to tissue damage.	(45, 46)

### 4.4 Future perspective

Recent advancements have significantly improved our understanding of the dynamic regulatory mechanisms of autophagy in periodontal disease. Autophagy plays a vital role in periodontal protection by eliminating pathogens, degrading inflammasomes, and maintaining mitochondrial homeostasis, thereby alleviating oxidative stress and inflammation (46, 47). However, excessive autophagy activation can result in lysosomal dysfunction, exacerbate oxidative stress responses, and promote osteoclast-mediated bone resorption. Critical molecular regulators, including the PINK1-Parkin pathway (48), RAB7A-mediated autophagosome maturation, and USP4 deubiquitinating modification (40), have emerged as essential targets for modulating the “double-edged sword” effects of autophagy. Regarding innovative therapies, exosomes derived from mesenchymal stem cells have shown potential in promoting periodontal regeneration through the delivery of regulatory molecules (49). Furthermore, interdisciplinary research has uncovered molecular links between systemic diseases, such as diabetes and oral cancer (50), and disruptions in periodontal autophagy, offering new perspectives for developing future precision therapies.

Looking ahead, several research areas are critical for fully harnessing the potential of autophagy in periodontal disease regulation:

Therapeutic Regulation: There is an urgent need to develop novel small-molecule drugs, gene-editing technologies, and localized delivery systems capable of precisely controlling autophagic activity. Such approaches could not only prevent pathogens from exploiting autophagy for immune evasion but also enhance cellular repair and tissue regeneration.

Diagnostic Methods: Future research should focus on developing bioassays and imaging techniques that are based on autophagy-related biomarkers. These methods could facilitate the early detection of periodontal tissue damage and changes in autophagic status, thereby providing a foundation for personalized therapeutic interventions.

Tissue Regeneration: Autophagy plays a critical role in tissue repair and regeneration in periodontal disease (PD). By enhancing autophagic processes in periodontal ligament stem cells and osteoblasts, bone regeneration and tissue healing could be accelerated. Biomaterials that activate autophagy, along with gene therapies targeting autophagic pathways, have significant potential for regenerating damaged periodontal tissues. The integration of stem cell therapies with advanced biomaterials, coupled with autophagy modulation, could establish innovative regenerative platforms, significantly advancing tissue repair and functional recovery in periodontal bone and soft tissues.

However, insufficient investment in interdisciplinary integration, clinical translation, and large-scale data analysis may hinder rapid progress in this field. Enhanced funding and resource allocation, along with deeper integration of basic and clinical research, are essential for elucidating the precise mechanisms by which autophagy regulates periodontal disease and promoting its widespread clinical application, ultimately accelerating the translation from mechanistic insights to precision medicine.

## 5 Strengths and limitations

### 5.1 Strengths

In recent years, there has been a significant increase in the volume of publications concerning periodontal disease (PD) and autophagy. In this context, this study provides a comprehensive bibliometric analysis of the relationship between autophagy and periodontal disease, synthesizing the existing body of knowledge using English-language publications indexed in the Web of Science. Specifically, it identifies 20 key terms with substantial citation bursts, thereby illuminating critical research trends and emerging hotspots within the field. Additionally, by offering a clear overview of influential authors, journals, and key research trajectories, this study provides valuable insights into the current state of research. A notable strength of this analysis is the inclusion of a “Future Perspective” section, which examines the potential applications of autophagy in therapeutic modulation, diagnostics, and tissue regeneration for the management of periodontal disease. Furthermore, the use of rigorous bibliometric tools ensures the reliability and reproducibility of the findings, while the study’s clinical relevance bridges the gap between academic research and practical applications. This underscores the translational potential of autophagy in PD treatment and regenerative medicine.

### 5.2 Limitations

In line with other bibliometric analyses, this study also has several limitations: (1) CiteSpace cannot fully replace systematic searches; (2) The study primarily focused on English-language publications, which may have excluded valuable research published in other languages. Future studies could incorporate non-English publications to offer a more comprehensive global perspective; (3) This study was confined to publications from 2006 to 2023, which may have led to the exclusion of significant research. Future studies could expand the review to include publications prior to 2006, offering a more comprehensive understanding of the evolution of autophagy-related research in periodontal disease. (4) Data were obtained from the WoSCC database, which is commonly used in scientometric analyses. However, the analysis could have been further enhanced by incorporating additional databases. Future studies should broaden the search to encompass a wider range of literature; (5) A limitation of citation-based analysis is its tendency to underrepresent recent high-impact studies due to citation time lags. Recently published high-quality studies may be excluded due to insufficient citation counts, which can affect the identification of research hotspots and emerging trends. Additionally, articles with significant contributions may not have accumulated sufficient citations to fully reflect their impact. Future studies should track citation trends over time to better understand the long-term impact of recent publications.

Despite these limitations, we believe this study effectively captures the hotspots and emerging trends in the role of autophagy in PD. Based on this study, future researchers can gain a comprehensive understanding of the current research landscape and select research directions based on the identified hotspots and emerging trends.

## 6 Conclusion

In recent years, the role of autophagy in periodontal diseases (PD) has attracted significant attention, as demonstrated by the increasing number of related publications. Through a bibliometric analysis of research on autophagy and periodontal diseases, this study offers a comprehensive overview of research trends, key findings, and the potential impact of autophagy on the pathogenesis and treatment of periodontal diseases. Over the past 18 years, the number of publications and citations in this field has steadily increased, indicating growing interest and research enthusiasm among scholars. China is the leading country in this research worldwide. Moving forward, it is crucial to enhance collaboration and communication among institutions and authors. Key research hotspots in this field include apoptosis, Porphyromonas gingivalis, oxidative stress, inflammation, periodontitis, osteogenic differentiation, cell death, and orthodontic tooth movement. Additionally, exploring how to harness the protective functions of autophagy for managing periodontal diseases may emerge as a key research direction. This study summarizes the knowledge base in this field, highlights current research hotspots and emerging trends, and offers valuable guidance for future researchers in selecting appropriate research directions.

## Data Availability

The original contributions presented in this study are included in this article/Supplementary material, further inquiries can be directed to the corresponding authors.

## Footnotes

1
https://www.webofscience.com
